# Phosphorus diffusion gettering process of multicrystalline silicon using a sacrificial porous silicon layer

**DOI:** 10.1186/1556-276X-7-424

**Published:** 2012-07-31

**Authors:** Derbali Lotfi, Ezzaouia Hatem

**Affiliations:** 1 Photovoltaïc laboratory, Research and Technology Center of Energy, Technopôle de Borj-Cédria. BP 95, Hammam-Lif, 2050, Tunisia

**Keywords:** Multicrystalline silicon, Porous silicon, Defect density, Grain boundaries, Gettering, WTC-120 lifetime tester

## Abstract

The aims of this work are to getter undesirable impurities from low-cost multicrystalline silicon (mc-Si) wafers and then enhance their electronic properties. We used an efficient process which consists of applying phosphorus diffusion into a sacrificial porous silicon (PS) layer in which the gettered impurities have been trapped after the heat treatment. As we have expected, after removing the phosphorus-rich PS layer, the electrical properties of the mc-Si wafers were significantly improved. The PS layers, realized on both sides of the mc-Si substrates, were formed by the stain-etching technique. The phosphorus treatment was achieved using a liquid POCl_3_-based source on both sides of the mc-Si wafers. The realized phosphorus/PS/Si/PS/phosphorus structures were annealed at a temperature ranging between 700°C and 950°C under a controlled O_2_ atmosphere, which allows phosphorus to diffuse throughout the PS layers and to getter eventual metal impurities towards the phosphorus-doped PS layer. The effect of this gettering procedure was investigated by means of internal quantum efficiency and the dark current–voltage (*I*-*V*) characteristics. The minority carrier lifetime measurements were made using a WTC-120 photoconductance lifetime tester. The serial resistance and the shunt resistance carried out from the dark *I*-*V* curves confirm this gettering-related solar cell improvement. It has been shown that the photovoltaic parameters of the gettered silicon solar cells were improved with regard to the ungettered one, which proves the beneficial effect of this gettering process on the conversion efficiency of the multicrystalline silicon solar cells.

## Background

Due to the high price of fossil energy source and huge pollution of environmental issue, in the recent years, the development of renewable energy has regained our attention. The development of the solar energy industry is one of the most popular technologies in renewable energy. The majority of solar cells are made from crystalline silicon; more than half of the crystalline silicon solar cell production is based on multicrystalline silicon (mc-Si). The quality of multicrystalline silicon (mc-Si) wafers may become worse in the future for several reasons; the main reason is that lower-quality feedstock will probably be used for cost reduction and availability reasons [1]. Improving conversion efficiency using a low-cost material development is the main research activity in the photovoltaic field [2,3]. Several experiments demonstrated that the electrical properties of crystalline silicon solar cells can be improved by coating the n + emitter region with a porous silicon layer [2-5], and many technologies have been used for this purpose [6-8], such as using vanadium oxide as an antireflection coating [9]. The possibility of improving the electrical properties of silicon wafers, by extracting impurities from them, using thermal treatment under oxygen atmosphere, or phosphorus diffusion, is well known [10-13]. Besides, the porous silicon (PS) layer may be used as an efficient sacrificial layer for gettering metallic impurities [11,14]. There are two general classifications of gettering, namely extrinsic and intrinsic. Extrinsic gettering refers to gettering that employs external means to create the damage or stress in the silicon lattice in such a way that extended defects needed for trapping impurities are formed. These chemically reactive trapping sites are usually located at the wafer surfaces, away from the bulk. Intrinsic gettering is using oxygen to enforce precipitation of metallic impurities, such as copper, not at the wafer surface but in its bulk. A preparatory step in this case is a thermal treatment causing precipitation of the excess oxygen which, for a variety of reasons related to the specific properties of oxygen in silicon, forms oxygen precipitates in the bulk of the wafer rather than at its surface. Those oxygen precipitates then act as gettering sites for metallic impurities. PS may also be used as an excellent antireflective coating or as a passivating layer on the emitter of crystalline silicon [15]. Most often, the formation of PS can create defects in the surface of the Si substrate that may enhance the gettering effect as extrinsic gettering effect. A phosphorus gettering model was proposed [16], which stated that the gettering speed is controlled by two steps. The first step is to limit the gettering temperature by releasing diffusion of metal impurities, and the second step is to control the best gettering temperature by the segregation function. Other authors [17] proposed that silicon self-interstitial current generated during phosphorus (P) diffusion is an essential factor of the gettering mechanism, and also found that Fermi level-enhanced solubility in the P diffused layer contributes to the gettering effect. The use of an infrared (IR) furnace for the fabrication of p-n junction [18,19] and gettering impurity through the porous silicon layer [20] is one of the processes that may be used to reach this goal. In this work, we will investigate the increase of carrier lifetime and the conversion efficiency of multicrystalline silicon solar cells gettered by a phosphorous gettering process using a sacrificial porous silicon layer in which the impurities will be trapped.

## Methods

The starting material was a p-type (boron-doped) multicrystalline silicon substrate, 400 μm thick with a resistivity of 1 to 2 Ω cm. To avoid changes in grains and grain barriers (GBs) from wafer to wafer, samples were selected from consecutive mc-Si wafers sharpened successively in the same ingot. Similar wafers, which were vertically adjacent to each other in the ingot, were used to compare and confirm the gettering effect. Therefore, differences between them, after different processing, can be interpreted as being due to variations in the process parameters rather than to material variations. PS layers were formed on both sides by the stain-etching technique using HF/HNO_3_/H_2_O solution with a 1:3:5 volume composition [3]. A POCl_3_/acetone liquid source was used for the diffusion process [20]. We optimized the POCl_3_/acetone ratio to 1:5. The POCl_3_ spreading out was realized by the spinning technique onto p-type multicrystalline silicon wafers. After drying at 200°C for solvent evaporation, the realized P/PS/Si/PS/P structure undergoes a heat treatment in an IR furnace under an O_2_ atmosphere. The temperature annealing was varied in the range of 700°C to 950°C for 60 min. This heat treatment has been applied in order to allow P diffusion throughout the PS layer. After annealing the samples, the phosphorous-doped region (the phosphorous-doped PS layer) was removed from both sides using a chemical etching (HF 16%: HNO_3_ 64%:CH_3_COOH 20%) solution. In order to perform the solar cell fabrication process, the n+/p junction was achieved using a simple phosphorus diffusion technique. The back aluminum (Ag/Al) and the front Ag contacts were screen printed using AMI Presco CP885 screen printer (Affiliated Manufacturers, Inc., North Branch, NJ, USA) and fired at 850°C and 620°C, respectively. The gettering effect has been evaluated by measuring the minority carrier lifetime, the dark and illuminated *IV* characteristics, and the defect density at the GBs of the mc-Si substrates. The *IV* measurements under illumination have been performed using PASAN cell tester CT 801 (PASAN Measurement Systems, Neuchâtel, Switzerland). The effective minority carrier lifetime (*τ*_eff_) in the mc-Si substrates was measured using a WTC-120 photoconductance lifetime tester (Sinton Instruments, Boulder, CO, USA) under the quasi-steady-state lifetime measurement using the generalized analysis condition.

## Results and discussion

**Figure 1:**
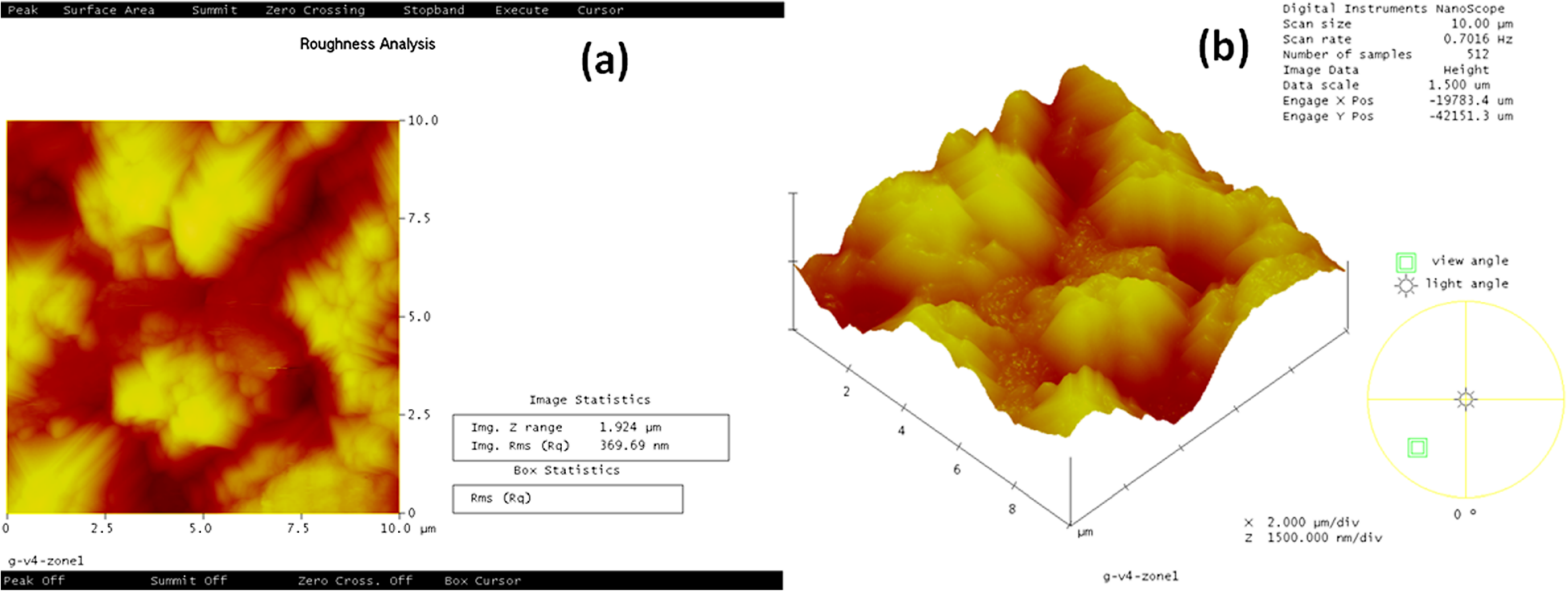
**AFM topography of the porous silicon layer formed by the stain-etching technique. (a)** Porous silicon formed on both sides of the samples. **(b)** 3D AFM topography before treatment with phosphorous.

The porous silicon layer created onto the front and back surfaces of the multicrystalline silicon wafers has a great importance in this work because it has the role of an external trapping site of impurities extracted from the samples. Figure 1a,b presents an atomic force microscopy (AFM) topography of the porous silicon layer formed by the stain-etching technique.

The porous silicon structure has been treated with phosphorus, followed by heat treatment at various temperatures ranging between 700°C and 950°C. Then, the treated PS layers were removed, and as a result, purified mc-Si substrates have been obtained. To confirm this gettering effect on the electrical properties of the samples, we have investigated the variation of the effective minority carrier lifetime.

The effective minority carrier lifetime of the gettered mc-Si wafers has been measured under the quasi-steady-state lifetime measurement using the generalized analysis condition. The measured area always includes some grain boundaries and thus provides an overall picture of both the intra-grain material and the extra recombination at grain boundaries. It is a combination of mechanisms that determines the performance of practical solar cells. The surfaces of the wafer are always present; we have reduced their possible contribution, but especially for the highest lifetimes measured, they are still likely to have a significant effect. The lifetimes reported here should be considered as *effective lifetimes*, which include both surface and volume recombination components. In many practical cases, there may be several sources of recombination in a sample, such as recombination through impurities in the wafer bulk or recombination at the surfaces. The effective lifetime represents the combined impact of all of these competing recombination channels. The effective lifetime *τ*_eff_ measured under low-level injection at Δ*n* = 1 × 10^14^ cm^-3^ was taken for the calculations under generalized conditions. For 2 Ω cm (the resistivity of the wafers) and N_D_ = 5 × 10^14^ cm^-3^, the wafers satisfy the low-level injection condition (Δ*n* < <N_D_).

The effectiveness of this gettering process is confirmed by the increase of the minority carrier lifetime, measured at the same carrier density, before and after treatment. Table 1 summarizes the effect of this treatment process on the effective minority carrier lifetime (*τ*_eff_). We have obtained a very high lifetime value of the minority carrier after a thermal treatment at 900°C. We notice that *τ*_eff_ increases when the annealing temperature increases, as shown in Table 1.

**Table 1:** **Measured effective lifetime *τ***_**eff **_**under low-level injection**

**Temperature (°C)**	***τ***_**eff **_**(μsec)**
Reference	2.92
700	37.17
800	59.76
850	83.16
900	90.88
950	13.07

*τ*_eff_, effective minority carrier lifetime.

Multicrystalline silicon incorporates many impurities and defects that limit the minority carrier lifetime and, thus, the solar cell performance [21]. This significant variation of the minority carrier lifetime would indicate that a non-negligible quantity of unwanted impurities has been gettered and removed from the Si material and a decrease of the grain and GBs carrier recombination activities. Considering that the measurement area includes several grain boundaries, the results presented here indicate a very low recombination activity at the grain boundaries when comparing treated wafers to untreated one (reference).

It is important to note that the surface and volume recombination mechanisms coexist. We considered that both front and back surfaces of the wafer are identical since both of them have been subjected to the same treatment. The equation that takes into account all the recombination mechanisms that are present in the wafer is given by [22]:

(1) $$\frac{1}{\tau_{\text{eff}}}=\frac{1}{\tau_{\text{bulk}}}+\frac{2S}{W}.$$

Supposing that both front and back surfaces of all samples were subjected to the same passivation effect and have the same surface state, we can neglect the effect of the surfaces [22,23]. Consequently, (*τ*_bulk_) can be deduced:

(2) $$\frac{1}{\tau_{\text{eff}}}\propto \frac{1}{\tau_{\text{bulk}}}.$$

Therefore, we can determine the bulk recombination properties of the wafer from the effective minority carrier lifetime measurements. As a result, the obtained improvement indicates that impurities in the bulk of the treated wafers have been removed, and the recombination activities have been decreased noticeably.

**Figure 2:**
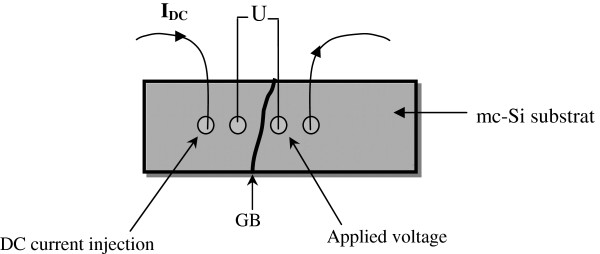
**Schematic illustration of the dark *I*-*V *measurements at a selected grain boundary.**

It is now interesting to investigate the variation of the defect density at the GBs while changing the treatment conditions, which is a one of the important parameters to evaluate the effect of our gettering process. We have performed four aluminum ohmic contacts on two adjacent grains (as shown in Figure 2). The aluminum was deposited by screen printing using Al/Ag containing aluminum and a small amount of silver in order to ensure the welding of the contact’s edges. A DC current passes through the selected GBs between two adjacent grains using two external contacts. Using the two inner contacts, we have measured the voltage *U*. Figure 2 shows a schematic illustration of the dark *IV* measurements across four selected GBs to evaluate the defect density (*N*_B_) [24].

The zero-bias conductance for grain boundary is defined by [24,25]:

(3) $$\sigma_{B}=\frac{J_{\text{DC}}}{U}$$

where *U* is the applied voltage. It can be written as [26]:

(4) $$\sigma_{B}=\frac{1}{R_{S}}=\frac{e^{2}}{KT}N_{\text{a}}\nu_{t}(1-C)\exp(-\frac{V_{\text{B}}}{KT})$$

The GB potential barrier can be written as following:

(5) $$V_{\text{B}}=KT\;\text{Log}\left[\frac{eAN_{a}S}{N_{v}\sigma_{B}KT}\right]$$

where *K* is the Boltzmann constant, *T* is the ambient temperature, *e* is the elementary charge, *A* is the Richardson constant, and *S* is the grain boundary surface crossed by the current flow. *N*_v_ and *N*_a_ are the effective densities of the valence states and the doping concentration, respectively.

The defect density N_B_(cm^-2^) was determined as a function of the GB potential barrier *V*_B _[27]:

(6) $$N_{B}=\frac{Q_{B}}{e}=\frac{\sqrt{32\varepsilon_{\text{γ}}\varepsilon_{0}N_{\text{a}}V_{B}}}{e}.$$

**Figure 3:**
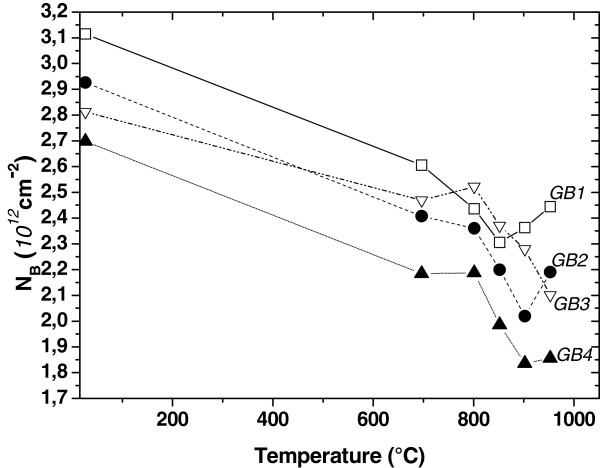
Effect of annealing the PS/phosphorus structure on the defect density at four selected GBs.

Identical wafers have been treated and identical GBs in the gettered samples show that defect density decreases when samples were subjected to thermal treatment, as shown in Figure 3. This significant variation of the defect density at the GBs would indicate that a non-negligible quantity of undesirable impurities have been gettered and removed from the grain boundaries. Thus, the recombination activities at the GBs have been reduced noticeably. These improvements could be attributed to the removal of eventual bulk metal impurities (Fe, Cu, etc.) and their trapping into the n^+^/PS layer. We notice that high-temperature annealing in infrared furnace has enhanced the impurity diffusion into the sacrificial porous silicon layer. Thus, the phosphorus-rich PS acts as an efficient external gettering site in which the impurities are captured due to the high thermal treatment. The phosphorus diffusion into grain boundaries, at least in the region near the front and back surfaces, can explain the considerable decrease of the defect density and the recombination activities into the GBs, as shown in Figure 3.

**Figure 4:**
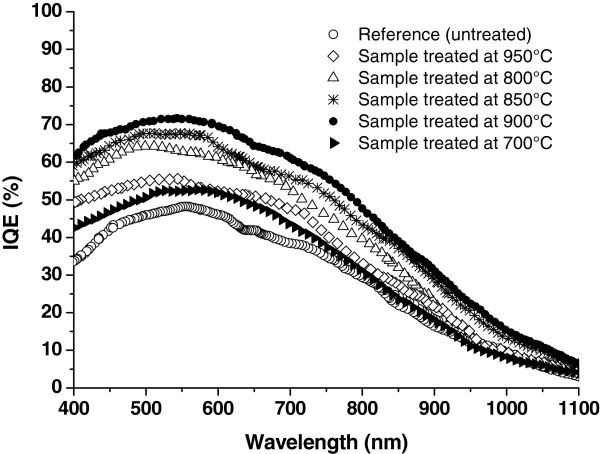
The internal quantum efficiency dependence with the thermal treatment.

To understand the impact of this gettering process on cell performance, the internal quantum efficiency (IQE) measurements were evaluated at various annealing temperatures. All annealing temperatures exhibit an increased blue response in short wavelengths (400 to 750 nm), which is due to an important decrease of the surface recombination velocity and an improvement of the carrier collection at the emitter region, as shown in Figure 4.

A significant increase of the IQE in the long-wavelength range 700 to 1,000 nm (red response) is observed. This improvement can be explained by the important reduction of the carrier recombination activities in the bulk of the treated wafers, which is proven by the obtained minority carrier lifetime values in Table 1, and the significant reduction of the defect density at the GBs, at least in the region near the front and back surfaces. The observed behavior of the spectral response indicates that our gettering process leads to an efficient surface and bulk passivation and indicates an extended effect deep into the bulk of the substrate, which we suggest to be considered especially at the grain boundaries. The gettered solar cell at 900°C shows the highest IQE, which is not surprising because the effective lifetime of the minority carrier in the wafer treated at 900°C proved to be the highest.

**Figure 5:**
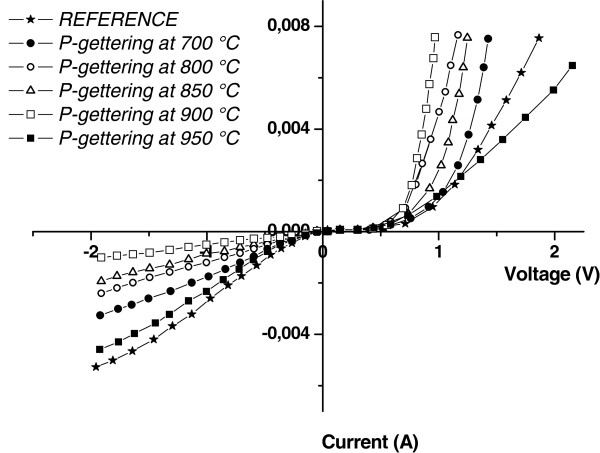
**Dark *I*-*V *characteristics of the untreated and the PS-gettered samples at 700°C, 800°C, and 900°C.**

The dark *I*-*V* characteristics have been investigated in Figure 5. After PS treatment, a significant improvement in the typical dark *I*-*V* characteristic was observed: the leakage current has considerably decreased, which is due to the reduction of the impurity concentration by gettering undesirable metallic impurities and defects that diffuse at getter sites present at the pore walls of the phosphorous-rich PS layer. The *I*-*V* characteristics clearly show a significant improvement of the rectifying behavior and a noticeable decrease of the reverse current after treatment, indicating an efficient passivation of grain and grain boundaries of the mc-Si wafers.

In multicrystalline silicon solar cells, the saturation current is essentially due to short circuiting via GBs. The current–voltage characteristic of the gettered samples shows an enhancement of the saturation current, which is a sign of the passivation effect of this gettering process at GBs. From these dark *IV* curves, we determined the series resistance (*R*_s_) [28] and the shunt resistance (*R*_sh_) of the gettered and untreated cells.

**Figure 6:**
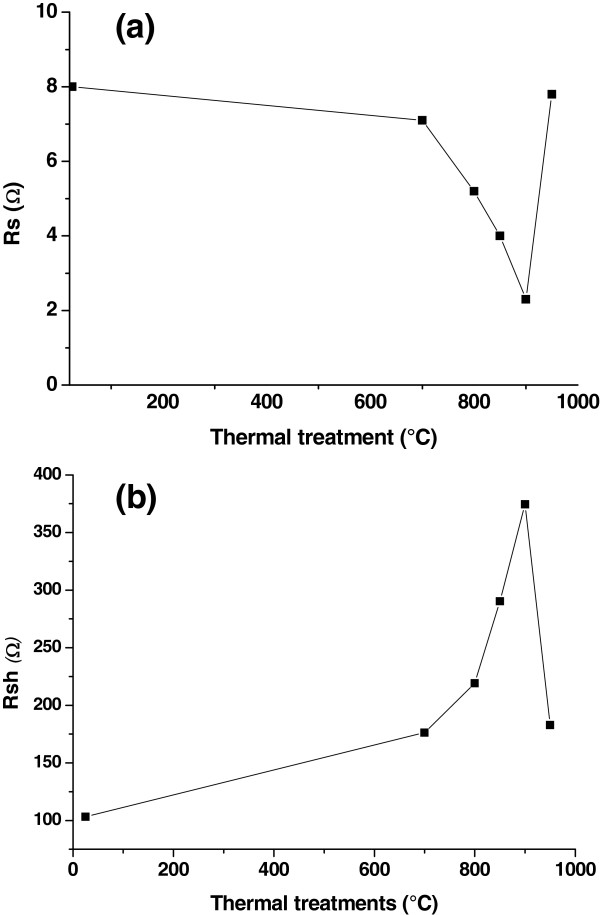
**Series (*R***_**s**_**) (a) and shunt (*R***_**sh**_**) (b) resistances of the gettered multicrystalline solar cells.**

Figure 6 depicts the variation of the series and shunt resistances. We notice a significant increase of the shunt resistance (*R*_sh_) and a diminution of the series resistance (*R*_s_). The improvement of *R*_sh_ can be attributed to an efficient passivation of the grain and GBs of the mc-Si wafers and the gettering of unwanted impurities.

However, the enhancement of *R*_s_ could be due to the gettering effect in adjacent grains [29]. The deep recombination centers present at the GBs and bulk defects have been reduced, and an important decrease of the surface recombination velocity has been obtained. Gettering at 950°C seems to have a bad effect on the electrical properties of the cells, which could be due to the deep diffusion of phosphorus into the mc-Si substrate when annealing the sample at 950°C.

**Figure 7:**
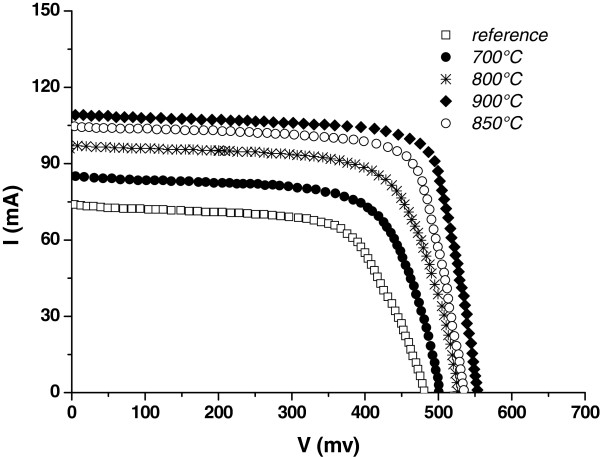
***I*-*V *characteristics under AM1.5 illumination of the gettered multicrystalline solar cells.**

In Figure 7, we carried out the *I*-*V* characteristic measurement of obtained solar cells under AM1.5 illumination (100 mW/cm^2^). Four parameters of solar cells were used to define illuminated solar cells: the short-circuit current (*I*_sc_), the open-circuit voltage (*V*_oc_), the fill factor (FF), and the efficiency (*η*).

The optimal temperature is about 900°C. This optimum could be the result of the competition between the release of impurities from the bulk and a capture of impurities in the gettering layer, which has been removed after the thermal treatment. Below the optimum temperature, gettering process is limited by the release or the diffusion [28,30] of metallic impurities towards the gettering layer. This behavior is confirmed by the degradation of the *IV* characteristics for the samples treated at temperatures exceeding the optimum one.

Table 2 shows the variation of the *I*_sc_ and the *V*_oc_ after phosphorous gettering at different temperatures. Our gettering process improves the *I*_sc_ from 74 to 109 mA (sample gettered at 900°C) and the *V*_oc_ from 0.48 to 0.55 mV (sample gettered at 900°C). The effect of gettering mc-Si using the combination of phosphorous diffusion into a sacrificial PS layer and a thermal treatment becomes apparent when looking at the different cell parameters. The untreated solar cell (reference sample) shows a drastically lower short-circuit current density (*J*_sc_) compared to the cells subjected to the above treatment, especially when annealed at 900°C. The same tendency can be stated for the open-circuit voltage (*V*_oc_), which is 70 mV higher in the case of the gettered solar cell at 900°C, compared to the reference wafer (untreated). We conclude that phosphorous gettering process under O_2_ atmosphere, using a sacrificial PS layer on both sides, improves the electrical parameters and the performances of the cells via the reduction of the carrier recombination activities. This leads to an enhancement of the short-circuit current and the open-circuit voltage and an increase of the conversion efficiency of the mc-Si solar cells.

**Table 2:** Comparison of the electrical parameters of phosphorous gettered and ungettered multicrystalline silicon solar cells

**Temperature (°C)**	***J***_**sc **_**(mA/cm²)**	***V***_**oc **_**(V)**	**FF**	***η***
Reference	18.5	0.48	67	5.9
700	21.3	0.50	69.4	7.4
800	24.4	0.52	70.5	8.9
850	26.3	0.53	74.8	10.4
900	27.4	0.55	77.2	11.6

FF, fill factor; *J*_sc_, short-circuit current density; *V*_oc_, open-circuit voltage; *η*, efficiency.

## Conclusions

The application of a sacrificial porous silicon layer, followed by a deposition of thin POCl_3_ liquid film and then subjecting to a thermal treatment, has been proven to be able to getter undesirable impurities from the mc-Si substrate. The best results were achieved after gettering at 900°C for 60 min. This gettering process has led to a significant increase of the minority carrier lifetime and a noticeable decrease of the defect density at grain boundaries. The improvement of the internal quantum efficiency confirms the beneficial effect of the above treatment. Impurities and defects at the surfaces and at the GBs have been trapped inside the P-doped PS layer after the heat treatment. Obtained results show a significant improvement of the electrical performances of the mc-Si solar cells, and the conversion efficiency has been increased from 5.9% to 11.6% in the sample gettered at 900°C.

## Competing interests

The authors declare that they have no competing interests.

## Authors' contributions

DL carried out the synthesis and analysis of the experiment, fabricated the porous silicon layers, measured the minority carrier lifetime and performed the AFM imaging and IQE and *I-V* measurements of the gettered solar cells. DL and HE contributed to the conception and design of the experiments, data interpretation and writing of the manuscript, discussed the results, contributed to the manuscript text, commented on the manuscript and approved its final version. Both authors read and approved the final manuscript.
